# Influence of Antimony Oxide on Epoxy Based Intumescent Flame Retardation Coating System

**DOI:** 10.3390/polym12112721

**Published:** 2020-11-17

**Authors:** Riyazuddin, Samrin Bano, Fohad Mabood Husain, Rais Ahmad Khan, Ali Alsalme, Jamal Akhter Siddique

**Affiliations:** 1 Department of Food Science and Nutrition, King Saud University, Riyadh-11451, Saudi Arabia; samriyaz5@gmail.com (R.); fhusain@ksu.edu.sa (F.M.H.); 2 Center of Chemical Sciences and Technology, Institute of Science and Technology, JNTU, Hyderabad 500085, Telangana State, India; samrinbano384@gmail.com; 3 Department of Chemistry, College of Science, King Saud University, Riyadh-11451, Saudi Arabia; krais@ksu.edu.sa; 4 Department of Chemistry, Aligarh Muslim University, Aligarh 202002, India

**Keywords:** modified ammonium polyphosphate, antimony oxide, synergist, Epoxy coatings

## Abstract

Ethylenediamine modified Ammonium polyphosphate (EDA-MAPP), and Charring-Foaming Agents (CFA) was prepared via a simple chemical approach and further utilizes for the preparation of Epoxy resin based intumescent flame retardation coatings. The ratio belongs to MAPP and CFA was fixed at 2:1 ratio. Comparative thermo gravimetric analysis TGA study of Modified Ammonium polyphosphate (MAPP) and Ammonium polyphosphate (APP) investigated. Sb_2_O_3_ was introduced into flame retardation coating formulation at various amounts to evaluate the synergistic action of Sb_2_O_3_ along with flame retardant coating system. The synergistic action of Sb_2_O_3_ on flame retardation coating formulation was studied by vertical burning test (UL-94V), thermo gravimetric analysis (TGA), Limited Oxygen Index (LOI), and Fourier Transform Infra-Red spectroscopy (FTIR). The UL-94V results indicated that adding Sb_2_O_3_ effectively increased flame retardancy and meets V-0 ratings at each concentration. The TGA results revealed that the amalgamation of Sb_2_O_3_ at each concentration effectively increased the thermal stability of the flame retardant coating system. Cone-calorimeter study results that Sb_2_O_3_ successfully minimized the combustion parameters like, Peak Heat Release Rate (PHRR), and Total Heat Release (THR). The FTIR result shows that Sb_2_O_3_ can react with MAPP and generates the dense-charred layer which prevents the transfer of heat and oxygen.

## 1. Introduction

Wood has been using as primary structural raw material due to its valuable properties, such as complexion, low mass, high strength, and low-level insulation properties. Due to its high flammability, the application of wood is limited in some fields for the purpose of safety [1,2,3]. Polymer-fabricated materials are recognized now a days as the major engineering material that involve in aerospace, automobile, shipbuilding, construction, and many other multiple fields are linked with people’s routine lives. Such materials show strength and low weight ratio along with decent stability in terms of physical and chemical behavior, their resistance capacity in terms of corrosion, heat, chemical, etc. with self-lubrication and other superior properties is quite acceptable [4,5,6].

Flammability of polymers is a big challenge in implement and expands their uses; scientists have established many approaches to overcome with this problem, like as flame retardant filler technology, fire retardant coating technology, fire retardant solution soaking and chemical grafting flame retardant technology [7,8,9]. Flame retardant coating is one of the solutions to overcome with flammability, majorly divided into two categories: (1) Intumescent flame retardant coatings and (2) Non-intumescent flame retardant coatings. The most useful intumescent coatings are more preferred than non-intumescent coating because intumescent coatings forms a foam like structure on the material surface that restricts the flow heat and oxygen. So, the practical application of intumescent flame retardant (IFR) coating technology is recognized as more suitable for reducing the fire damages of wood [10]. Generally, IFR system consist three major ingredients: (1) acid source, (2) carbonizing agent, and (3) foaming agent [11]. For the period of heating, IFR coating mechanism can form the protective charred layer with foam to protect the wood substrate from burning process [12]. The char can be formed by cross-link and cyclization reaction of flame retardant molecules [13]. This charred layer slows down the shifting of oxygen and heat in between substrate and its surroundings [14].

Previous studies demonstrated that pentaerythritol (PER) and ammonium polyphosphate (APP) combination has shown impressive intumescence flame retardation [15,16,17]. Some other studies also reported that APP, PER, and Melamine were used in the composition of IFR system, but it was not given satisfactory outputs due to its low-level performance of anti-oxidation and fire retardancy [18]. PER act as carbonization source but it is moisture sensitive in nature. So it can easily absorb the water content from atmosphere and ooze out from the IFR system, leading to a terrible corrosion. Several studies have been done to develop and their practical application of different types of carbonizing agents [19]. In this regard, from the last decade researchers have been showing a great attention towards triazine derivate to synthesize highly stable charring-foaming agents [20]. Triazine derivative acts as a charring agent and also as foaming agent as they contain abundant amount of carbon and nitrogen atoms in its structure [21,22]. Different kinds of Triazine derivatives have been utilizes as foaming and charring agent in IFR systems and their effects were extensively investigated [23,24]. There are so many studies where they used APP and Triazine derivates as an intumescent flame retardant system. However, it has some limitations, because triazine derivatives produce the char which show poor efficiency. In order to improve char thickness and hardness, need to incorporate synergistic agents in IFR system. There are some studies in which they have added synergistic agents, such as zeolites [25], organoboran siloxanes [26], and some metal compounds and transition metal oxides. Such studies clearly explain that synergistic agents can greatly improve the thickness of the char by forming cross linkages.

Antimony trioxide (Sb_2_O_3_) is a white colored powder, non-carcinogenic agent. It can be involved as a synergist to improve the performance of an intumescent flame retardant system. It is very cheap and environmentally friendly. It considered as a flame retardant during the condensation phase. While burning, it can react with hydroxyl groups of the ammonium polyphosphate causing to endothermic decomposition.

The current study, an enthusiastically approaches, cost effective, and non-hazardous antimony trioxide selected as a synergistic agent to investigate its action on Epoxy/IFR coating system. The IFR method consisting of ethylenediamine modified ammonium polyphosphate and charring foaming agent (CFA). In this work, APP is modified with ethylenediamine because APP behaves as acid source and blowing agent, on other hand MAPP can show charring action too. CFA polymer was prepared from triazine molecule. It is hydrophobic in nature and has thermally stable triazine ring. Due to these reasons, CFA cannot be affected by atmospheric moisture and show great flame retardancy. For the first time, we attempted a trial to prepare a new IFR coating composition of MAPP and CFA to improve thermal strength and flame retardant performance of the coating composition. To this coating composition, an eco-friendly synergistic agent Sb_2_O_3_ is added to increase the thickness of the intumescent char.

The limited oxygen index (LOI), vertical burning test (UL-94V), thermo-gravimetric analysis (TGA), Fourier transform infra-red spectroscopy (FT-IR), and cone calorimeter studies have been employed to investigate the synergism between antimony trioxide and Epoxy/IFR system.

## 2. Materials and Experimental

The Epoxy resin and amine hardener used in this study were obtained from Sigma Aldrich (Munich, Germany). Ammonium polyphosphate (APP, Crystalline form II, *n* ≥ 1000) and Ethanol (95% pure) were obtained from Samchun pure chemicals, South-korea. Ethylenediamine (EDA, Purity: 99.5%), acetone (Pure: 99%), ethanolamine (ETA, Pure: 99%), and antimony tri-oxide (powder, 5 μm, ReagentPlus^®^, 99%) were analytical grade and obtained from Sigma Aldrich. Cyanuric chloride (Purity-99%) was procured by Sigma Aldrich. All the chemicals and solvents were used as received.

### 2.1. Preparation of MAPP

The clean and dry three-neck round bottom flask of 1000 mL which is equipped along a magnetic stirrer, a mixer of ethanol and water (400 mL:15 mL) was poured. At 30 min later, 9 g of ethylenediamine was poured into the flask and stirred at 200 rpm for 20 min. Then 50 g of APP was added slowly into the above solution. The prepared solution was heated around 80 °C for 6 h. After the completion of reaction, the obtained mixture gets cooled at room temperature. The resulted white solid was filtered, washed with ethanol and water. The obtained white solids were kept in hot air oven at 50 °C for next 20 h to remove moisture content.

### 2.2. Preparation CFA

The derivative of triazine-containing macromolecule that was designated as charring-foaming agent (CFA) synthesized via nucleophillic substitution reaction.

In the initial step, 1 mol of cyanuric chloride and 500 mL of acetone were poured into a dry and clean three-neck 2000 mL, flask already fitted with a magnetic stirrer which was immersed in ice water bath. Then 1 mol of each ethanolamine and NaOH were completely dissolved in distilled water, and the mixture was slowly added into the flask for a period of 2 h. This step was maintained at 0–10 °C for 4 h.

The second step of the reaction takes 0.5 mol of ethylenediamine and 1 mol of NaOH were dissolved in distilled water and the solution was added drop wise to the above reaction mixture. Then the reaction temperature gets increased to 50–60 °C. This reaction was permissible to continue for 4 h.

In the third step of chemical reaction, again a mixture of 1 mol of NaOH and 0.5 mol of ethylenediamine were dissolved in distilled water; this aqueous solution was added slowly into the reaction flask. Then the reaction temperature was upraised to 75 °C to evaporate acetone solvent. This step was allowed to proceed for 4 h. when the reaction was completed; the obtained mixture was cooled at room temperature and filtered. After filtration, the collected particles of white solid were washed with distilled water and dried in hot air oven at 60 °C for 3 h.

### 2.3. Sample Preparation

Intumescent flame retardant (IFR) coating system contained of MAPP, CFA and a Sb_2_O_3_ as a synergist. The proportion of MAPP to CFA was set at 2:1 ratio and the incorporation of Sb_2_O_3_ changes from 0 to 4 wt% respectively. The intumescent flame retardant coating formulations are represented in Table 1. All the constituents were homogeneously mixed using high-speed disperse mixer to prepare IFR coating formulations. The prepared coatings were implied on particular sized plywood pieces manually by using a paintbrush at room temperature. This process was repeated to make 1.5 ± 2 mm coating thickness. Once coating has been done the samples were dried for 48 h at room temperature.

### 2.4. Characterization Methods

#### 2.4.1. FT-IR Test

A Nicolet Avator 360 spectrometer instrument (Nicolet, Madison, WI, USA) was involved for FTIR analysis in the range of 4000–500 cm^−1^. For the FT-IR analysis, the samples were pressed into pellet forms with KBr powder.

#### 2.4.2. X-Ray Diffraction Study

D8 X-ray Diffractometer model with Cu Kα radiation were used for the XRD study for the structural analysis of samples. The scanning rate of 0.02° per second with the 2 degrees and the scanning range was 5–50°.

### 2.5. Flame Retardancy Test

The LOI values of all coating formulations were measured as per ASTM- D2863 standard procedure on HC-2C Oxygen Index instrument (Jiangning County Analytical Instrument Factory, Jiangning, China). The coating formulations were applied on the plywood sheets of 130 mm × 6.5 mm × 3 mm dimension to perform the LOI test.

The prepared intumescent flame retardant coatings were applied on the surface of all sides of plywood pieces of the dimension of 127 mm × 27 mm × 3 mm. Then the coated plywood pieces were dried at room temperature for 48 h. The UL-94V test was performed as per ASTM-D3801 standard method. The coated plywood sheets were ignited for 30 s to calculate the burning rate, and flame retardation times of each sample.

### 2.6. Thermo Gravimetric Analysis Test

Thermal stability of the all coating formulations was performed by using SDT Q600 V20.9 Build-20 instrument (TA Instruments, New Castle, DE, USA) with 20 °C/min of heating rate at a temperature range of 30–800 °C under nitrogen atmosphere at the flow rate of 20 mL/min. The weight of each coating formulation was taken approximately 4–5 mg.

### 2.7. Combustion Test

The combustion parameters of all coating formulations were performed on cone calorimeter in accordance with ISO 5660–2002 standard method by exposing 50 kW m^−2^ of external heat flux. All samples were vertically laid on sample holder.

## 3. Results and Discussion

### 3.1. FTIR of MAPP

**Figure 1:**
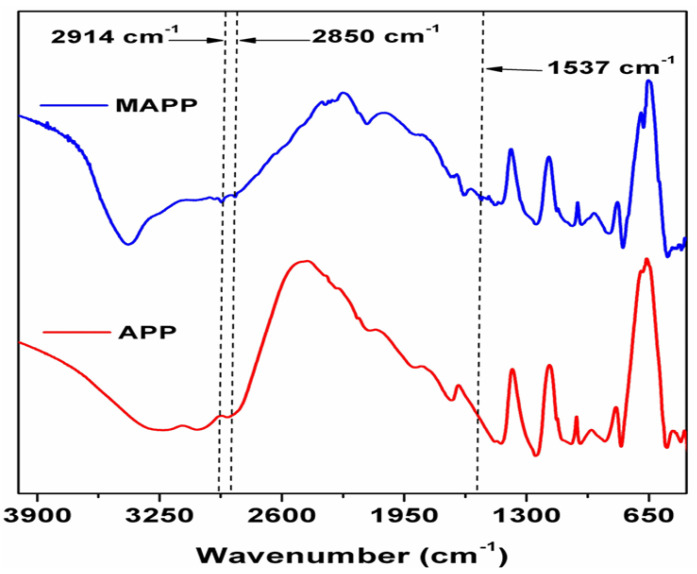
FT-IR spectra of Modified Ammonium polyphosphate (MAPP) and Ammonium polyphosphate (APP).

The Figure 1 shows, FTIR spectra of MAPP and APP. The peak attributed at 3407 cm^−1^ is because of the NH_4_^+^ ion asymmetric stretching vibrations [27]. From this data, it can be seen that MAPP spectrum contains peaks at 2915, 2864, and 1535 cm^−1^, which specifically attributed for the characteristic stretching absorption peaks of -CH_2_-CH_2_- and NH_3_^+^ ion. The appeared peaks which are corresponding to -CH_2_-CH_2_- and NH_3_^+^ ion in MAPP proved that NH_3_^(+)(−)^ O-P bond is formed successfully.

### 3.2. XRD of MAPP

**Figure 2:**
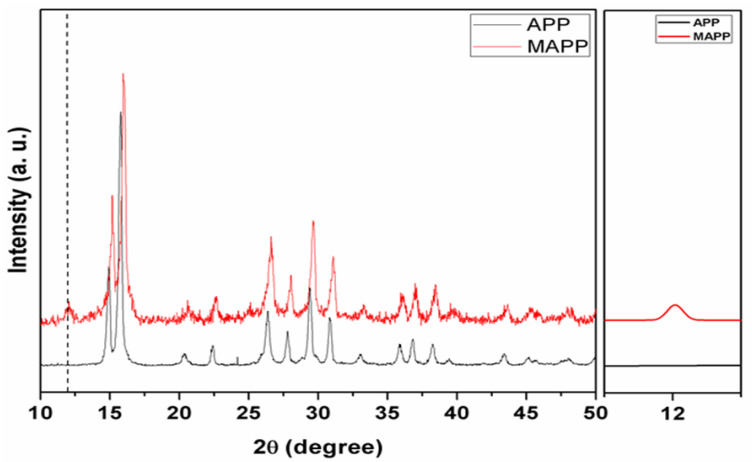
The XRD curves of APP and MAPP.

Figure 2 shows the XRD spectra of APP and MAPP. The diffraction peaks of APP and MAPP are appeared at same position, but a new peak at 11.92° is obtained for MAPP. This specific peak in MAPP is attributed for the presence of (NH_4_)_5_P_3_O_10_·H_2_O. This result showing that the crystalline structures of APP are unchanged by the reaction between APP and ethylenediamine.

### 3.3. TGA Graph of MAPP and APP

**Figure 3:**
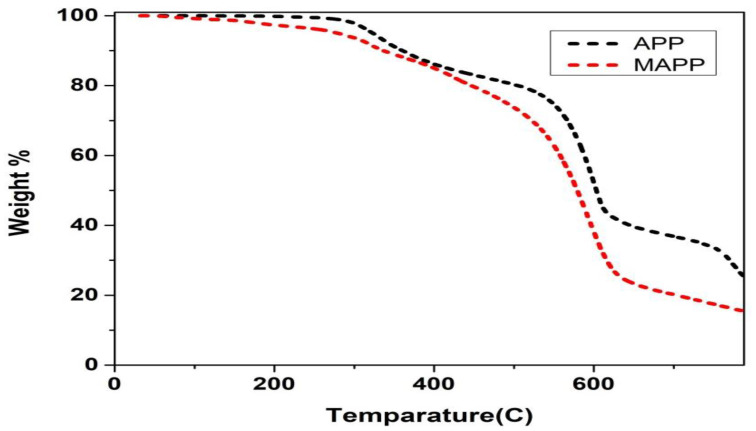
TGA curve of APP and MAPP.

The thermal degradation curves of APP and MAPP shows under N2 atmosphere in Figure 3. Their degradation behaviors had apparent differences. For APP, there were two decomposing processes which located at about 315 °C and 640 °C, respectively. However, MAPP shown only one peak during its degradation process. The thermal degradation process of APP could be divided into two steps. The first step was in the range of 200–410 °C, it might be due to the elimination of ammonia and water content in the thermal degradation process of polyphosphate. The second step was beyond 450 °C, this weight loss was attributed to the release of phosphoric acid, polyphosphoric acid, with APP decomposition. The thermal degradation of MAPP is same as thermal degradation of APP, and also included the thermal behavior of EDA salt. After incorporating the EDA, the residue of MAPP was much higher than that of APP between 300 and 400 °C. The formation of more char residue at this stage contributed to the following formation of intumescent char layer, so it can be observed that the introduction of EDA in APP effectively enhanced the flame retardancy in the combustion tests.

### 3.4. FTIR of CFA

**Figure 4:**
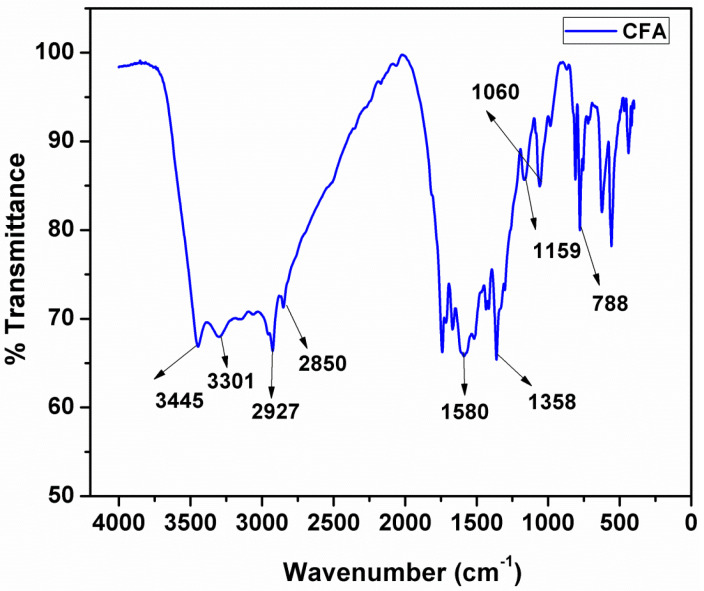
FT-IR curve of and charring foaming agent (CFA).

The FTIR spectrum of CFA is shown in Figure 4. This spectrum consist the broad adsorption peaks between the range of 3300–3450 cm^−1^ are because of symmetrical stretching vibrations of O-H and N-H bonds; and the peaks at 2850 cm^−1^ and 2927 cm^−1^ can be allocated to ν_C-H_ in -CH_2_-CH_2_- group. The peaks at 1580, 1358, 1159 and 1060 cm^−1^ can be assigned to the ν_C = N_ ν_tr-N,_ ν_C-N_ and ν_C-O_, respectively. Moreover, this spectrum does not show the peak at 850 cm^−1^ that is corresponding to the C-Cl stretching vibration and it indicates that CFA is formed successfully.

### 3.5. Thermo-Gravimetric Analysis (TGA)of CFA

The losses in mass of sample with respect to increase in temperature can be evaluated by thermo-gravimetric analysis and it gives the information that the char have been formed; this is the degradation behavior and thermal stability of the sample. 

**Figure 5:**
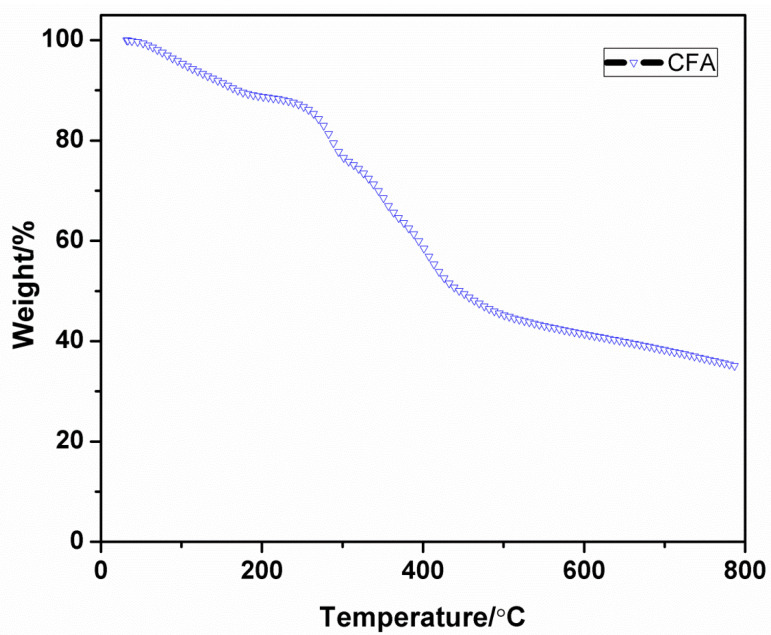
TGA curve of CFA.

**Figure 6:**
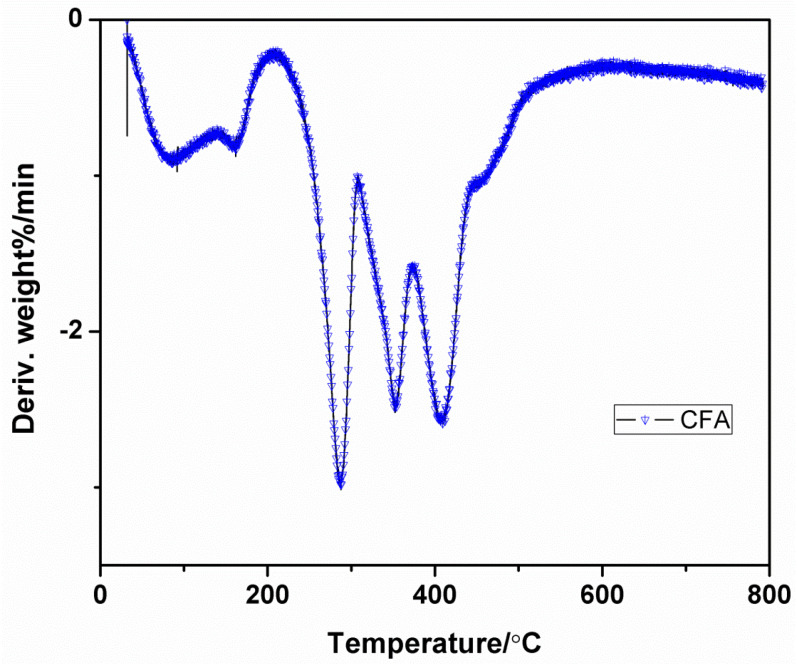
Differential Thermal Analysis (DTG) curve of CFA.

The Differential Thermal Analysis (DTA) and TGA curves of the CFA are represented in Figure 5 and Figure 6 with Table 2. The CFA has shown a great amount of char at 700 and 800 °C are 38.20 and 34.80, respectively. The result states that the synthesized CFA is thermally more stable and admirable charring-foaming agent at high temperature because of its stable triazine ring structure. In the DTG curve, it can be clearly seen that the CFA decomposition has three main steps. The initial step take place in the range of 250–310 °C and the peak at 287 °C is due to the evaporation of ammonia and nitrogen gas from the CFA. The second degradation range appears at the range from 310 to 370 °C, this has a peak at 353 °C and it is attributed for the partial bond breakage. The third step of decomposition occurs at 370 to 432 °C temperature range. At this step, the weight loss has occurred due to the thermal decomposition macromolecular backbone structure.

### 3.6. Thermo Gravimetric Analysis of Coatings

**Figure 7:**
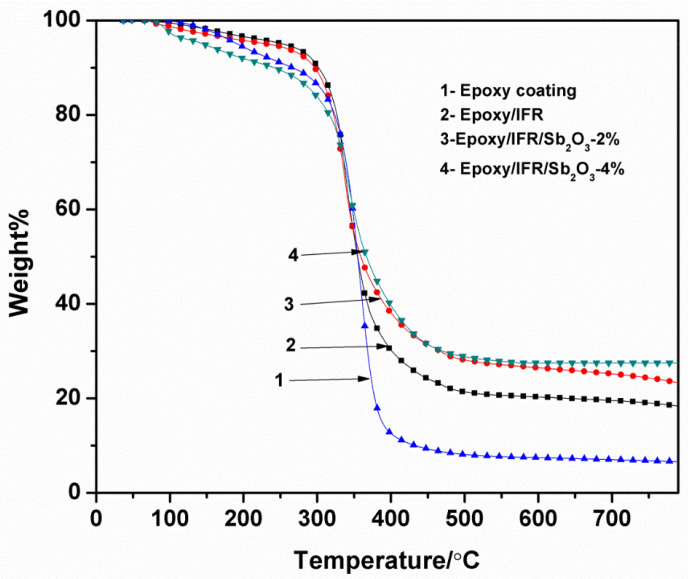
TGA curves of coatings.

**Figure 8:**
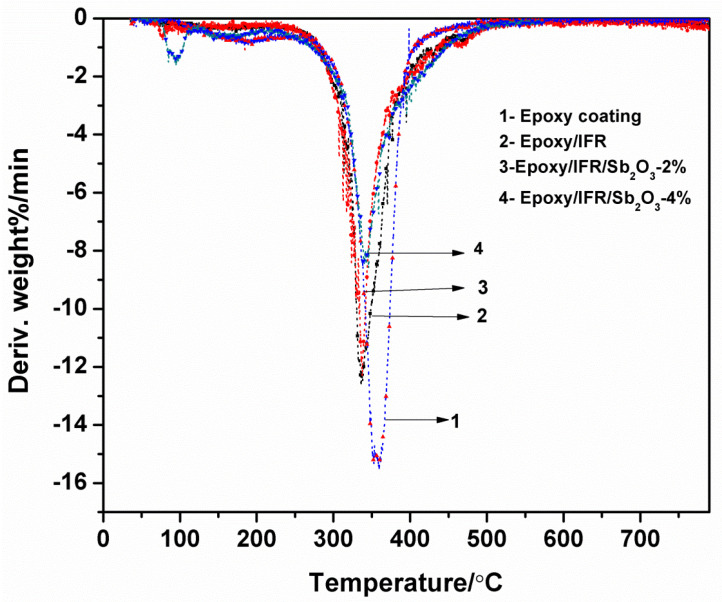
DTG curves of coatings.

Figure 7 and Figure 8 and Table 3 show the TGA results of the IFR coatings with various amounts of Sb_2_O_3_ under nitrogen atmosphere. In comparison, the TGA curves of the Epoxy/IFR/Sb_2_O_3_-2% and Epoxy/IFR/Sb_2_O_3_-4% are showing high thermal stability. From the data, it can be observed that the pure resin decompose at 192 °C (5% Tonset) with a maximum temperature of 359 °C where it has 15.5 MLR%/min. Pure resin coating has shown 6.6% char residue at above 500 °C and it is burnt up to 93.5%.

The T_Onset_ 5% temperature of pure Epoxy, Epoxy/IFR, Epoxy/IFR/Sb_2_O_3_-2% and Epoxy/IFR/Sb_2_O_3_-4% coatings are 192, 187, 165 and 122 °C respectively. The char residue percentage at 800 °C of the Epoxy, Epoxy/IFR, Epoxy/IFR/Sb_2_O_3_-2% and Epoxy/IFR/Sb_2_O_3_-4% coatings are 6.6, 18.5, 23.7, and 27.5% with MLR%/min values of 15.2, 12.3, 11.1, and 8.3 respectively. From this data it can be found that Epoxy/IFR is good thermal stable than pure Epoxy coating because during burning MAPP and CFA undergo esterification reaction to form a char which protects the flow of oxygen and heat to the coating material therefore it will be more stable.

The Table 3 data clearly indicates that Epoxy/IFR/Sb_2_O_3_-2% and Epoxy/IFR/Sb_2_O_3_-4% coatings have less thermal initial decomposition temperature, less MLR%/min and high char residue percentages. The reason is due to that the Sb_2_O_3_ can catalyze the esterification reaction between MAPP and CFA to form a thick protective char layer. This CFA is gently involved in esterification reaction because it is a polyhydroxyl triazine polymer.

From these data, it is clear that addition of Sb_2_O_3_ can upsurge the thermal stability of the coating formulations.

### 3.7. Flame Retardancy Test

Table 4 represents the values of LOI of the Epoxy/IFR systems with varied ratio of MAPP and CFA. The data clearly show that by increasing addition of MAPP, the LOI values first remarkably increased, then gradually decreased. When the ratio of MAPP and CFA is 2:1, it reached to maximum value at 31.2 and this one pass the burning test with V-0 rating. Because a condensation reaction take place in between MAPP and CFA to form a dense, protective char layer which can inhibit the flow of oxygen and heat in between burning material and its surroundings. In the case of less content of MAPP, a crumbled charred layer is formed which is ineffective to restrict the flow of heat and oxygen. In the other hand, when the amount of MAPP reached maximum level, it could then produce an excess amount of gasses, such as ammonia and nitrogen, which resulted in broken of the stable charred layer. Hence, the combination of MAPP and CFA has shown effective action at optimal ratio of 2:1.

Table 5 represents the LOI values and UL-94V ratings of Epoxy/IFR and Epoxy/IFR/Sb_2_O_3_ coating systems. The data clearly reveal that the addition of Sb_2_O_3_ effectively increases the LOI value of Epoxy/IFR system. The addition of Sb_2_O_3_ at 2 wt%, 4 wt%, remarkably increased the LOI values from 31.2 to 31.9 and 32.6, respectively. The reason behind to increase the LOI values is that Sb_2_O_3_ can involve in a chemical reaction with IFR system. In addition to this, Sb_2_O_3_ can also act as catalyst for the condensation reaction between MAPP and CFA.

From the Table 5 data, it can be known that Epoxy/IFR and Epoxy/IFR/Sb_2_O_3_ systems pass the UL-94V test and can reach V-0 rating. The IFR system form the intumescent char on burning which is responsible to slow down the burning process and finally results in the extinguish of fire. Thus, all the coating formulation can reach V-0 rating.

### 3.8. Compositions of Charred Layers

Previous studies reveal that upon burning, intumescent flame retardant system forms a protective charred layer on the surface of the material [28,29,30]. The charred layer can inhibit the flow of heat and oxygen around the material and surroundings. The IFR system also releases the non-flammable gases and moisture to the surroundings to dilute the heat and oxygen around the burning material. Consequently, the char layer can stop the burning process and increase the flame resistance of material.

The charred layers of all coating formulation formed by constant heating in the muffle furnace for 20 min at 500 °C.

**Figure 9:**
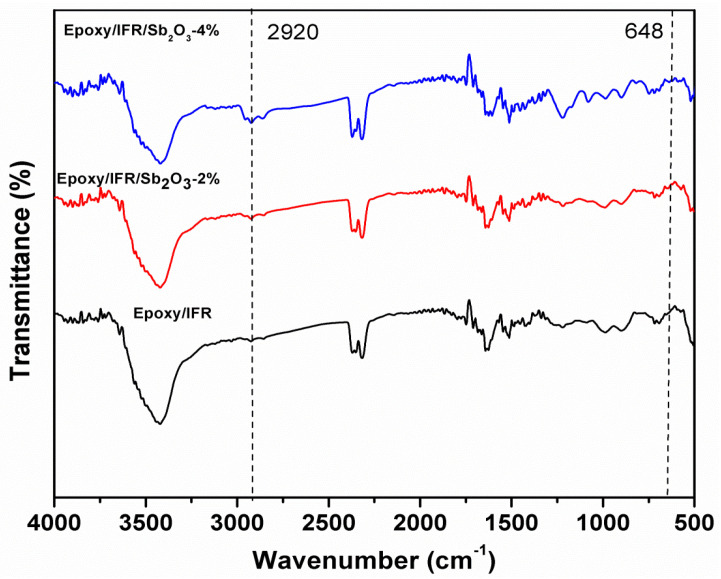
FTIR curves of Epoxy/IFR, Epoxy/IFR/Sb_2_O_3_-2%, Epoxy/IFR/Sb_2_O_3_-4% coatings.

Figure 9 shows the FT-IR spectrum of Epoxy/IFR, Epoxy/IFR/Sb_2_O_3_-2 wt%, and Epoxy/IFR/Sb_2_O_3_-4 wt% charred layers.

**Figure 10:**
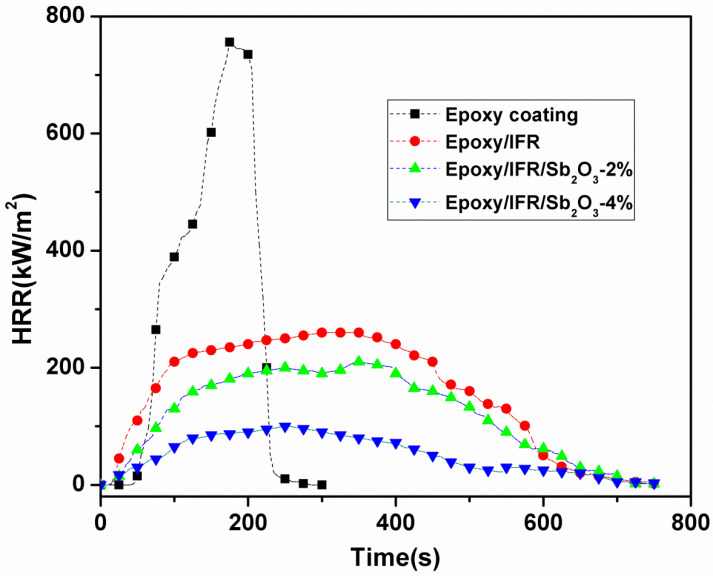
Peak heat release rate (PHRR) curves of coatings

From the Figure 10 data, it is clear that the absorption peaks in the range of 2900–2950 cm^−1^ corresponding to the symmetric and asymmetric stretching vibrations of C-H bonds in CH_2_ group.

The increasing intensity order of these peaks is Epoxy/IFR≤ Epoxy/IFR/Sb_2_O_3_-2 wt% ≤ Epoxy/IFR/Sb_2_O_3_-4 wt%. This order of absorption peak intensity demonstrates that the Sb_2_O_3_ can develop cross-linkages with IFR system and effectively protect the material form the fire. The peaks at 920–1088 cm^−1^ are assigned to the P-O-C, P-O-P, and P-O bonds stretching vibrations inside the phospho-carbonaceous complex. These peaks of Epoxy/IFR/Sb_2_O_3_-2 wt%, Epoxy/IFR/Sb_2_O_3_-4 wt%, are showing high intensity and broadness than Epoxy/IFR/Sb_2_O_3_-0 wt%. It indicates that it behaves as a catalyst, and show synergistic action on the establishment of a dense intumescent phsospho-carbonaceous char. The peak of absorption at 642 cm^−1^ is preferentially endorsed the stretching vibration of Sb-O bond which indicates that Sb_2_O_3_ can form cross-linkages with IFR system. The intensity of this peak increased as Sb_2_O_3_ amount increases. These results suggested that Sb_2_O_3_ can promote the thickness of the char.

### 3.9. Cone-Calorimetric Analysis

**Figure 11:**
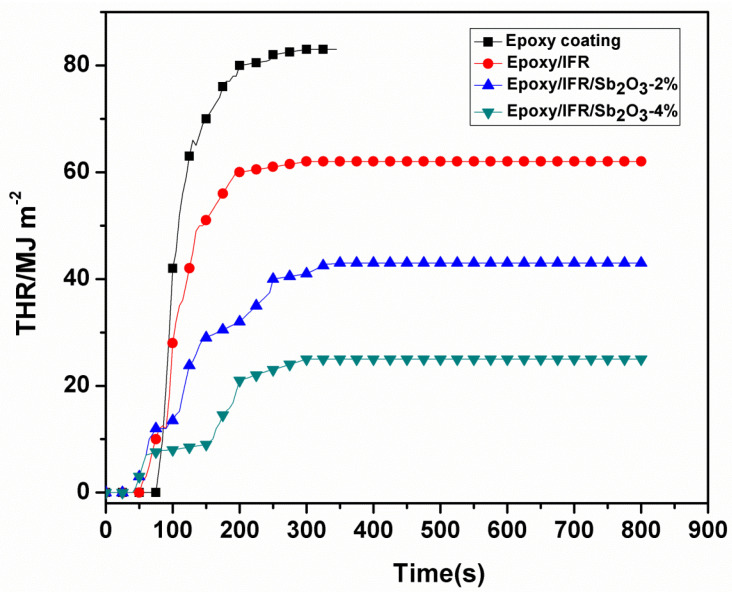
Total heat release (THR) curves of coatings.

The combustion study of a sample can be performed by cone calorimeter based on oxygen consumption principle. Cone calorimetric analysis provides the information for time to ignition (TTI), Peak heat release rate (PHRR), total heat release (THR), and total smoke production (TSP). The PHRR values are regarded as important indicator to characterize the flammability of a material in fire situation. The PHRR curves of all coating compositions are shown in Figure 10 and Table 6. Figure 11 and Table 6 indicates that pure Epoxy coating has shown a single and sharp HRR peak with 756 kW/m^2^ at 177 s, whereas the Epoxy/IFR, Epoxy/IFR/Sb_2_O_3_-2 wt%, and Epoxy/IFR/Sb_2_O_3_-4 wt%, coatings have PHRR peaks at 260, 207, 97.8 kW/m^2^, respectively. When the IFR and Sb_2_O_3_ are introduced into the coatings, a great decrease in PHRR values is observed. This is because, during the combustion process, the IFR system and Sb_2_O_3_ are involved in the development of a dense char layer that inhibits the flow of oxygen and heat to the material, thus lowing the intensity of the pyrolysis process and reducing the heat release quantity.

From the Figure 10 and Table 6 data, it can be seen that Epoxy/IFR, Epoxy/IFR/Sb_2_O_3_-2 wt% and Epoxy/IFR/Sb_2_O_3_-4 wt% coatings have cut the ignition time which are significantly endorsed to the formation of protective char layer. At the initial stage of burning, the temperature of these coatings rises gently because of the char formation; therefore, it results in shortening of ignition time. The Sb_2_O_3_ plays a remarkable role to improve the power of the char layer, protection of the char from damage and cracking to get low HRR values.

The THR values of all coatings are displayed in Figure 11 and Table 6. From the data, the difference in pure Epoxy coating and Epoxy/IFR, Epoxy/IFR/Sb_2_O_3_-2 wt%, Epoxy/IFR/Sb_2_O_3_-4 wt%, coatings is seen after 50s. The THR value of Epoxy is 82 MJ/m^2^ at 300s, whereas the THR value of Epoxy/IFR, Epoxy/IFR/Sb_2_O_3_-2 wt%, Epoxy/IFR/Sb_2_O_3_-4 wt%, are 63 MJ/m^2^, 42 MJ/m^2^, 24 MJ/m^2^ respectively. These results specify that addition of IFR and Sb_2_O_3_ greatly decreases the heat release rate. It is due to this that the MAPP and CFA agents form a dense protective char layer in the presence of Sb_2_O_3_ synergistic agent. The formed char layer lowers the heat releasing from the material.

From this data it is proved that Sb_2_O_3_ enhances the thickness and power of the char layer and improves the combustion properties of the coatings.

## 4. Conclusions

Ethylenediamine modified ammonium polyphosphate and N-ethanolamine triazine ethylenediamine copolymer (CFA) is synthesized successfully and their properties were thoroughly investigated and compared. Comparative TGA study of MAPP and APP also investigated. The synergistic effect of antimony trioxide on Epoxy/IFR coating system is evaluated. The UL-94V and LOI results indicate that Sb_2_O_3_ has significantly increased the flame retardancy of coating system. As the Sb_2_O_3_ content in the IFR coating system, the LOI values also increased remarkably. The TGA results provide hints about the addition of Sb_2_O_3_ greatly increasing the thermal steadiness of the coating system and the interaction between MAPP and Sb_2_O_3_ is well demonstrated by FTIR results. The study of synergistic effect of Sb_2_O_3_ on Epoxy/IFR coating system revealed the formation of stable protective charred layer. All the above results conclude that Sb_2_O_3_ is very effective synergistic agent in the Epoxy/IFR coating systems and helps in reducing the HRR emission remarkable. The study shows the good impact of Sb_2_O_3_ on epoxy based intumescent flame retardation coating system; still, further morphologically and mechanical study would give more information and open its role for further applications.

## Figures and Tables

**Table 1:** Composition of the intumescent flame retardation coatings.

Sample	Resin (%)	MAPP (%)	CFA (%)	Sb_2_O_3_
Pure Epoxy Resin	100	0	0	0
Epoxy/intumescent flame retardant (IFR)	70	20	10	0
Epoxy/IFR/Sb_2_O_3_-2%	68	20	10	2
Epoxy/IFR/Sb_2_O_3_-4%	66	20	10	4

**Table 2:** Thermal degradation data of CFA.

Sample	Char Residue (%)	
	700 °C	800 °C
CFA	38.20	34.80

**Table 3:** Thermo gravimetric results of the coatings under nitrogen atmosphere.

Sample	T_Onset_ (°C)	MLR%/min	Char Residue (%) at 800 °C
Pure Epoxy Resin	192	15.2	6.6
Epoxy/IFR	187	12.3	18.5
Epoxy/IFR/Sb_2_O_3_-2%	165	11.2	23.7
Epoxy/IFR/Sb_2_O_3_-4%	122	8.3	27.5

**Table 4:** Flame retardance results of Epoxy/IFR with different ratio of MAPP/CFA.

Epoxy (%)	Ratio of MAPP/CFA	LOI (%)	UL-94
100	0	25.2	No rating
70	1:0	26.5	V-1
70	1:1	27.1	V-1
70	1.5:1	28.3	V-0
70	2:1	31.2	V-0
70	3:1	29.6	V-2
70	4:1	28.4	V-2

**Table 5:** Limited Oxygen Index (LOI) and UL-94V results of the coatings.

Sample	LOI (%)	UL-94
Pure Epoxy Resin	25.2	No rating
Epoxy/IFR	31.2	V-0
Epoxy/IFR/Sb_2_O_3_-2%	31.9	V-0
Epoxy/IFR/Sb_2_O_3_-4%	32.6	V-0

**Table 6:** Cone calorimeter results of the coatings.

Sample	TTI (S)	PHRR (kW m^−2^)	THR (MJ m^−2^)
Pure Epoxy Resin	60	756	82
Epoxy/IFR	55	260	63
Epoxy/IFR/Sb_2_O_3_-2%	43	207	42
Epoxy/IFR/Sb_2_O_3_-4%	43	97	25

## References

[1] Nikolic M., Lawther J.M., Sanadi A.R. (2015). Use of nanofillers in wood coatings: A scientific review. J. Coat. Technol. Res..

[2] Khalifah A.S., Julien F., Shuyu L., Sabyasachi G. (2015). An Overview of Mode of Action and Analytical Methods for Evaluation of Gas Phase Activities of Flame Retardants. Polymers.

[3] Liu Q., Wang D., Li Z., Li Z., Peng X., Liu X., Zhang Y., Zheng P. (2020). Recent Developments in the Flame-Retardant System of Epoxy Resin. Materials.

[4] Dittenber D.B., Rao G. (2012). HVS: Critical review of recent publications on use of natural composites in infrastructure. Compos. A.

[5] Laoutid F., Bonnaud L., Alexandre M., Lopez-Cuesta J.-M., Dubois P. (2009). New prospects in flame retardant polymer materials: From fundamentals to nanocomposites. Mater. Sci. Eng. R Rep..

[6] Tawiah T., Yu B., Fei B. (2018). Advances in Flame Retardant Poly(Lactic Acid). Polymers.

[7] Bar M., Alagirusamy R., Das A. (2015). Flame Retardant Polymer Composites. Fibers Polym..

[8] Hirschler M.M. (2015). Flame retardants and heat release: Review of data on individual polymers. Fire Mater..

[9] Bourbigot S., Duquesne S. (2007). Fire retardant polymers: Recent developments and opportunities. J. Mater. Chem..

[10] Xiao Z., Liu S., Zhang Z., Mai C., Xie Y., Wang Q. (2018). Fire retardancy of an aqeous, intumescent and translucent wood varnish based on guanylurea phosphate and melamine-urea-farmaldehyde resin. Prog. Org. Coat..

[11] Alongi J., Han Z., Bourbigot S. (2015). Intumescence: Tradition versus novelty. A comprehensive review. Prog. Polym. Sci..

[12] Velencoso M.M., Battig A., Markwart J.C. (2018). Molecular firefighting—How modern phosphorus chemistry can help solve the challenge of flame retardancy. Angew. Chem..

[13] Chen J., Wang J., Chen H., Ni A., Ding A. (2020). Synergistic effect of intumescent flame retardant and attapulgite on mechanical properties and flame retardancy of glass fibre reinforced polyethylene composites. Compos. Struct..

[14] da Ribeiro S.P., Martins R.C., Barbosa G.M. (2020). Influence of the zeolite acidity on its synergistic action with a flame-retarding polymeric intumescent formulation. J. Mater. Sci..

[15] Khanal S., Zhang W., Ahmed S., Ali M., Xu S. (2018). Effects of intumescent flame retardant system consisting of tris (2-hydroxyethyl) isocyanurate and ammonium polyphosphate on the flame retardant properties of high-density polyethylene composites. Compos. Part A Appl..

[16] Evtushenko Y.M., Grigoriev Y.A., Rudakova T.A. (2019). Effect of aluminum hydroxide on the fireproofing properties of ammonium polyphosphate–pentaerythritol-based intumescent coating. J. Coat. Technol. Res..

[17] Badji A.M., Ndiaye D., Diallo A.K., Kebe N. (2016). The effect of Poly-ethylene-co-glycidyl methacrylate efficiency and clay platelets on thermal and rheological properties of wood polyethylene composites. Adv. Chem. Eng. Sci..

[18] Li G., Liang G., He T., Yang Q., Song W. (2007). Effects of EG and MoSi_2_ on thermal degradation of intumescent coating. Polym. Degrad. Stabil..

[19] Tang Q.H., Yang R.J., Song Y., He J.Y. (2014). Investigation of flame retarded thermoplastic poly (imide –urethane) with intumescent flame retardants. Ind. Eng. Chem. Res..

[20] Wu Z.H., Guo J.X., Fan Q.X., Wang Q., Cai Y.J. (2019). A comparison of thermal degradation kinetic mechanisms between elongational and shearing flow fields for nano-silica/IFR synergistic fire retardant polypropylene nanocomposites. Mater. Express.

[21] Dai J.F., Li B. (2010). Synthesis, thermal degradation and flame retardance of novel triazine ring containing macromolecules for intumescent flame retardant polypropylene. J. Appl. Polym. Sci..

[22] Li Y.T., Li B., Dai J.F., Jia H., Gao S.L. (2008). Synergistic effect of lanthanum oxide on novel intumescent flame retardant polypropylene system. Polym. Degrad. Stabil..

[23] Xu S., Zhang M., Li S.Y., Zeng H.Y., Tian X.Y., Wu K., Hu J. (2020). Intercalation of a novel containing nitrogen and sulfur anion into hydrotalcite and its highly efficient flame retardant performance for polypropylene. Clay Sci..

[24] Liu Y., Wang J.S., Deng C.L., Wang D.Y., Song Y.P., Wang Y.Z. (2010). The synergistic flame retardant effect of O-MMT on the intumescent flame retardant PP/CA/APP system. Polym. Adv. Technol..

[25] Cavdar A.D., Torun S.B., Ertas M., Mengeloglu F. (2019). Ammonium zeolite and ammonium phosphate applied as fire retardants for microcrystalline cellulose filled thermoplastic composites. Fire Saf. J..

[26] Wang Z., Liu Y., Li J. (2017). Regulating effects of nitrogenous bases on the char structure and flame retardancy of polypropylene/intumescent flame retardant composites. ACS Sustain. Chem. Eng..

[27] Nie S.B., Hu Y., Song L., He Q.L., Yang D.D., Chen H. (2008). Synergistic effect between a char forming agent (CFA) and microencapsulated ammonium polyphosphate on the thermal and flame retardant properties of polypropylene. Polym. Adv. Technol..

[28] Hansupo N., Tricot G., Bellayer S., Roussel P. (2018). Getting a better insight into the chemistry of decomposition of complex flame retarded formulation: New insights using solid state NMR. Polym. Degrad. Stab..

[29] Demir H., Arkìs E., Balkose D., Ulku S. (2005). Synergistic effect of natural zeolites on flame retardant additives. Polym. Degrad. Stabil..

[30] Marosi G., Márton A., Anna P., Bertalan G., Marosfoi B., Szép A. (2002). Ceramic precursor in flame retardant systems. Polym. Degrad. Stabil..

